# Non-Coding RNA Polymorphisms (rs2910164 and rs1333049) Associated With Prognosis of Lung Cancer Under Platinum-Based Chemotherapy

**DOI:** 10.3389/fphar.2021.709528

**Published:** 2021-09-16

**Authors:** Yi-Xin Chen, Juan Chen, Ji-Ye Yin, Hong-Hao Zhou, Bai-Mei He, Zhao-Qian Liu

**Affiliations:** ^1^ Hunan Key Laboratory of Pharmacogenetics, Department of Clinical Pharmacology, and National Clinical Research Center for Geriatric Disorders, Xiangya Hospital, Central South University, Changsha, China; ^2^ Institute of Clinical Pharmacology, Central South University, Changsha, China; ^3^ Departments of Pharmacy, Xiangya Hospital, Central South University, Changsha, China; ^4^ Departments of Gerontology, Xiangya Hospital, Central South University, Changsha, China

**Keywords:** polymorphism, lung cancer, non-coding RNA, prognosis, platinum-based chemotherapy

## Abstract

**Purpose:** Lung cancer is the largest cause of cancer deaths in the world. Platinum-based chemotherapy is a foundation of first-line chemotherapy. However, the prognosis of lung cancer treated with platinum-based chemotherapy is still a challenge. Single nucleotide polymorphism of non-coding RNA has the potential to be a biomarker, but its effectiveness has yet to be comprehensively assessed. In this study, we explored the association between polymorphisms of non-coding RNA and prognosis of lung cancer patients receiving platinum-based chemotherapy.

**Materials and Methods:** For 446 lung cancer patients receiving platinum-based chemotherapy, 22 single nucleotide polymorphisms of microRNA and long noncoding RNA were genotyped by MALDI-TOF mass spectrometry. Cox regression analysis, Kaplan-Meier method, and long-rank test have been performed to assess the association of overall and progression-free survival with polymorphisms.

**Results:** In the additive and dominant models, genetic polymorphism of *ANRIL* rs1333049 (G > C) was significantly associated with progression-free survival. Additive model: CC vs GC vs GG [HR = 0.84, *p* = 0.021, 95% CI (0.73–0.97)]; Recessive model: CC vs GG + GC [HR = 0.77, *p* = 0.026, 95% CI (0.61–0.97)]. In the dominant model, compared with the CC genotype patients, lower risk of death [HR = 0.81, *p* = 0.036, 95% CI (0.66–0.99)] and lower risk of progression [HR = 0.81, *p* = 0.040, 95% CI (0.67–0.99)] have been observed on the patients with CG or GG genotype in *miR-146A* rs2910164.

**Conclusion:** Our research demonstrated the potential of using *ANRIL* rs1333049 (G > C) and *miR-146A* rs2910164 (C > G) as biomarkers to support the prediction of a better prognosis for lung cancer patients receiving platinum-based chemotherapy.

## Introduction

Lung cancer is the most common and most morbid cancer in the world, with about 2,206,771 new cases and 1,796,144 deaths in 2020 (Sung et al., 2021). Lung cancer consists of two major categories, small cell lung cancer (SCLC) and non-small cell lung cancer (NSCLC). NSCLC, including squamous carcinoma, adenocarcinoma, large cell carcinoma, etc., accounts for more than 80% of the primary lung cancer cases (Herbst et al., 2018). In particular, from 2005 to 2014, the proportion of adenocarcinoma has gradually increased from 36.4 to 53.5%, making it the most common cause of primary lung cancer. Meanwhile, the proportion of squamous carcinoma has dropped from 45.4 to 34.4% (Shi et al., 2019). Compared to squamous carcinoma, adenocarcinoma has a worse prognosis. In addition, it is common to females and less related to smoking. The 5-years survival of lung cancer patients is low in-part due to advanced patient age and advanced cancer stage at diagnosis. Platinum-based chemotherapy is a foundation of first-line chemotherapy, and advances in targeted therapy and immunotherapy have not yet replaced its important role. Besides the traditional risk factors, such as age and status of clinical-stage, molecular biomarkers enable more accurate prediction model for the prognosis of lung cancer, providing molecular aspect insights to improve the treatment outcome of platinum-based chemotherapy.

Non-coding RNAs (ncRNA), including microRNAs (miRNAs), long noncoding RNAs (lncRNAs), circular RNAs (circRNAs), etc., cannot be translated into protein via transcription, therefore have been considered to be “junk” transcriptional products. However, recent studies have revealed the critical role of ncRNA regarding the cause and development of malignant tumors. Specifically, miRNAs are short non-protein-coding RNAs with ∼22 nt in length. MiRNAs could bind to 3′ untranslated region (3′-UTR) of protein-coding mRNA to regulate gene expression. lncRNAs, a class of non-coding transcripts over 200 base pairs, exert their biofunction via various regulatory mechanisms at the epigenetic, transcriptional, and post-transcriptional levels (Dykes and Emanueli, 2017). Additionally, lncRNAs can also act as the sponge for miRNAs to impair the function of miRNA (Veneziano et al., 2019).

Variants in non-coding intervals account for more than one-third of the variants identified by the genome-wide association studies and are related to certain diseases or phenotypes (Jazdzewski et al., 2008). Mutations in miRNA have an impact on its transcription, maturation and miRNA-mRNA interactions. (Ryan et al., 2010). Similarly, mutations of lncRNA also affect its structure, expression, and function (Pan et al., 2016). For this reason, a series of single nucleotide polymorphisms (SNPs) in miRNA and lncRNA have been reported to be potential prognostic markers.

Our previous studies have exhibited the finding that some of the selected polymorphisms of miRNAs and lncRNAs are associated with lung cancer susceptibility or platinum-based chemotherapy toxicity and response (Gong et al., 2016; Fang et al., 2017; Gong et al., 2017; Fang et al., 2018). This study further investigated the correlation between these SNPs and the prognosis of the lung cancer patients receiving chemotherapy.

## Materials and Methods

### Study Subjects

The samples used in this study were obtained from the Xiangya Hospital of Central South University as well as its Affiliated Cancer Hospital (both in Changsha, Hunan, China) between November 2011 and August 2014. 507 lung cancer patients from the ethnic group of the Han Chinese have been enrolled. The following inclusion criteria have been applied to filter the patients: 1. patients with the records of histopathological or cytological diagnosis of lung cancer. 2. patients receiving more than one cycle of platinum-based chemotherapy. Our exclusion criteria are: 1. patients receiving surgery, radiotherapy, or targeted therapy. 2. patients diagnosed with other malignancies. The last date of follow-up for this study is December 2020. The Response Evaluation Criteria (RECIST) guideline (ver. 1.1) has been used to determine the progression of disease during the follow-up. After filtering out the unqualified patients and the ones containing missing information, finally we identified 446 patients to conduct the following analyses.

All participants of this study have signed consent documents in written form. The study was conducted in accordance with the Declaration of Helsinki. The Ethics Committee of Central South University-Xiangya School of Medicine has approved our research with the registration number CTXY-110008–2. The Chinese Clinical Trial Registry has approved the clinical research admission of our study with the case ChiCTR-RO-12002873.

### Sample Collection and Processing, Genotyping

Peripheral whole blood samples (5 ml) were collected and stored in a −20°C environment. The genomic DNA was extracted using a Genomic DNA Purification Kit (Wizard Genomic DNA Purification Kit, A1620; Promega) according to the protocol. Genomic DNA samples were stored at −20°C until being used. We selected SNPs from two sources. On the one hand, we searched PubMed and Google Scholar for SNPs in ncRNA related to prognosis, chemotherapy efficacy and susceptibility of lung cancer. On the other hand, we searched SNP association studies regarding five prognosis-related lncRNAs (HOTAIR, H19, MEG3, HOTTIP, and ANRIL) (Supplementary Figure S1). The following selection criteria were applied to both of the groups in the screening process. First, we selected the SNPs that may be related to lung cancer prognosis and have limited research reports (occurrence ≤3). Meanwhile, these SNPs were also required to have a HR/OR value which significantly differs from 1. Second, after excluding the SNPs which have ≤5% MAF values, we sorted out the SNPs that meet at least one of the three conditions: 1. The Combined Annotation Dependent Depletion (CADD) score of the SNP is bigger than 15; 2. The SNP has biological functions demonstrated in previously reported experiments or predicted by databases; 3. The SNP is a tag SNP or it is located in the linkage disequilibrium (LD) region. The detailed information of the 22 selected SNPs is presented in Supplementary Table S1. Finally, 11 SNPs of miRNA and 11 SNPs of lncRNA have been selected to investigate. All genotyping performed in this study was conducted by the Sequenom MassArray Genotype Platform (Sequenom, San Diego, California, USA). Primers were designed by AssayDesigner (ver. 3.1). The sequences of primers are listed in Supplementary Table S2. The SNPs with >5% MAF and >95% call rate have been sorted out for analyses. The detailed information of the genotyped SNPs is shown in Table 1. The following databases were used: CADD score, miRNASNP v3, lncRNASNP2 (Miao et al., 2018; Rentzsch et al., 2019; Liu et al., 2021).

**TABLE 1:** The detailed information of the genotyped SNPs.

Gene	SNP ID	Chr	Position^a^	Call rate (%)	Polymorphism	MAF
*mir-196a-2*	rs11614913	12	53991815	98.43	C > T	0.46
*mir-146a*	rs2910164	5	160485411	96.41	C > G	0.48
*mir-5197*	rs2042253	5	143679868	99.33	T > C	0.40
*mir-378*	rs1076064	5	149732603	96.86	A > G	0.48
*mir-27a*	rs895819	19	13836478	98.21	T > C	0.30
*mir-149*	rs71428439	2	240456083	97.09	A > G	0.14
*mir-30c-1*	rs928508	1	40757742	98.88	G > A	0.50
*mir-605*	rs2043556	10	51299646	97.31	T > C	0.30
*let-7a-2*	rs629367	11	122146306	98.21	C > A	0.22
*mir-218–1*	rs11134527	5	168768351	97.76	G > A	0.41
*mir-499*	rs3746444	20	34990448	96.64	A > G	0.14
*H19*	rs2839698	11	1997623	99.10	G > A	0.30
*H19*	rs2107425	11	1999845	95.74	C > T	0.38
*MALAT1*	rs619586	11	65498698	97.76	A > G	0.09
*HOTAIR*	rs7958904	12	53963768	98.65	C > G	0.29
*HOTAIR*	rs4759314	12	53968051	97.76	G > A	0.14
*MEG3*	rs116907618	14	100857798	98.88	G > C	0.09
*HOTTIP*	rs3807598	7	27200863	96.41	C > G	0.49
*HOTTIP*	rs1859168	7	27202740	97.98	A > C	0.42
*CCAT2*	rs6983267	8	127401060	96.64	G > T	0.44
*ANRIL*	rs10120688	9	22056500	92.60	G > A	0.31
*ANRIL*	rs1333049	9	22125504	98.88	G > C	0.49

Chr, chromosome; MAF, minor allele frequency.

a Position, based on genome reference: GRCh38.

### Meta-Analysis

We searched PubMed and Google Scholar for up-to-date studies related to the prognosis of lung cancer (last accessed on 2021/8/28). Regardless of the treatment regimens, the researches involving OS or PFS were sorted out. And then two researchers on our team independently retrieved and documented the following information of each selected article, including name of the first author, publication year, ethnic origin of the population studied, population size of the study as well as HR (95%CI). We also used a random-effects model to estimate the overall HR. STATA 12.0 was used to conduct these analyses.

### Statistical Analysis

In this study, the Cox proportional hazard regression analysis has been used to appraise the influence of various clinical parameters on overall survival (OS) and progression-free survival (PFS). After being digitized, three genotypes of SNPs were imported into the additive, dominant, and recessive genetic models. And then, for the genotypes in these genetic models, the hazard ratio (HR) of OS and PFS, as well as the corresponding 95% confidence interval (95% CI), have been calculated based on the Cox proportional hazard model. Detailed demographic information, including age, sex, smoking status, histology, and status of clinical-stage, have been considered. The SNPs that are statistically significant have been further examined by the Kaplan-Meier curve and the log-rank test to assess appreciable differences of OS and PFS in patients with different genotypes. Afterward, stratification analysis was applied to evaluate the impact of different SNPs on various subgroups of patients. All the analyses listed above were performed by SPSS 26.0, and the forest plots were generated by the STATA 12.0 software. All the *p*-values mentioned in this report were two-sided, and *p* < 0.05 was deemed statistically significant.

## Results

### Clinical Parameters and Their Impact on Prognosis in Patients With Lung Cancer

The 446 qualified subjects included in this study all have the required demographic information. The distribution of these characteristics is presented in Table 2. The majority of the patients were males [348 (78.0%)], and the median age was 56 years, with a range of 21–80 years. The distribution of histology was divided into adenocarcinoma [201 (45.1%)], squamous cell carcinoma [159 (35.7%)], SCLC [55 (12.3%)], and other types. The majority of patients had a history of smoking [280 (62.8%)]. The patients in an advanced stage (NSCLC: stage III or IV; SCLC: extensive-stage disease) account for 92.4% of the subjects. After applying the cox proportional hazard regression analysis to evaluate the influence of these demographic factors on OS and PFS, the risk of death and progression for SCLC are higher compared with adenocarcinoma. Meanwhile, we have also observed that squamous cell carcinoma patients exhibited lower risk of death compared to adenocarcinoma (*p* = 0.044). It may be because that patients with squamous cell carcinoma were more likely to be diagnosed at early-stage and patients with all stages were recruited in our study. Additionally, the risk of death and progression among patients in the advanced stage are higher. In sum, most parameters listed above impact prognosis in a way similar to the findings of other studies. Other demographic factors of these lung cancer patients didn’t manifest notable association with the death risk or progression risk.

**TABLE 2:** Distribution of clinical characteristics and their impact on prognosis (*n* = 446).

			OS			PFS	
Variables	N (%)	MST (month)	HR (95% CI)	P Value	MST (month)	HR (95% CI)	P Value
**Total no. of patients**	446	—	—	—	—	—	—
**Age**	—	—	—	—	—	—	—
≤56	225 (50.4)	33.6	1.00	—	12.9	1.00	—
>56	221 (49.6)	31.7	1.05 (0.86,1.28)	0.609	11.2	1.08 (0.89–1.31)	0.454
**Sex**	—	—	—	—	—	—	—
Female	98 (22.0)	35.7	1.00	—	17.5	1.00	—
Male	348 (78.0)	30.2	1.15 (0.85–1.55)	0.380	10.8	1.03 (0.76–1.39)	0.842
**Histology**	—	—	—	—	—	—	—
Adenocarcinoma	201 (45.1)	34.0	1.00	—	13.4	1.00	—
Squamous carcinoma	159 (35.7)	42.0	0.79 (0.62–0.99)	0.044*	12.7	0.75 (0.60–0.95)	0.015*
Small cell carcinoma	55 (12.3)	15.3	1.98 (1.32–2.96)	0.001**	8.2	1.59 (1.08–2.34)	0.020*
Others	31 (7.0)	19.4	1.41 (0.96–2.08)	0.081	9.4	1.01 (0.68–1.48)	0.977
**Smoking status**	—	—	—	—	—	—	—
Never	166 (37.2)	34.9	1.00	—	16.0	1.00	—
Ever	280 (62.8)	28.8	1.10 (0.84–1.43)	0.499	10.3	1.15 (0.88–1.50)	0.304
**Clinical stage**	—	—	—	—	—	—	—
-II/LD	34 (7.6)	27.4	1.00	—	13.3	1.00	—
III-IV/ED	412 (92.4)	33.2	1.82 (1.13–2.94)	0.015*	11.7	1.92 (1.21–3.06)	0.006**

OS, overall survival; PFS, progression-free survival; LD, limited-stage disease; ED, extensive-stage disease; MST, median survival time; HR, hazard ratio; CI, confidence interval; *p < 0.05; **p < 0.01.

### Association of 22 Non-CodingRNA Single Nucleotide Polymorphisms With Prognosis in Lung Cancer Patients Treated With Platinum-Based Chemotherapy

**FIGURE 1:**
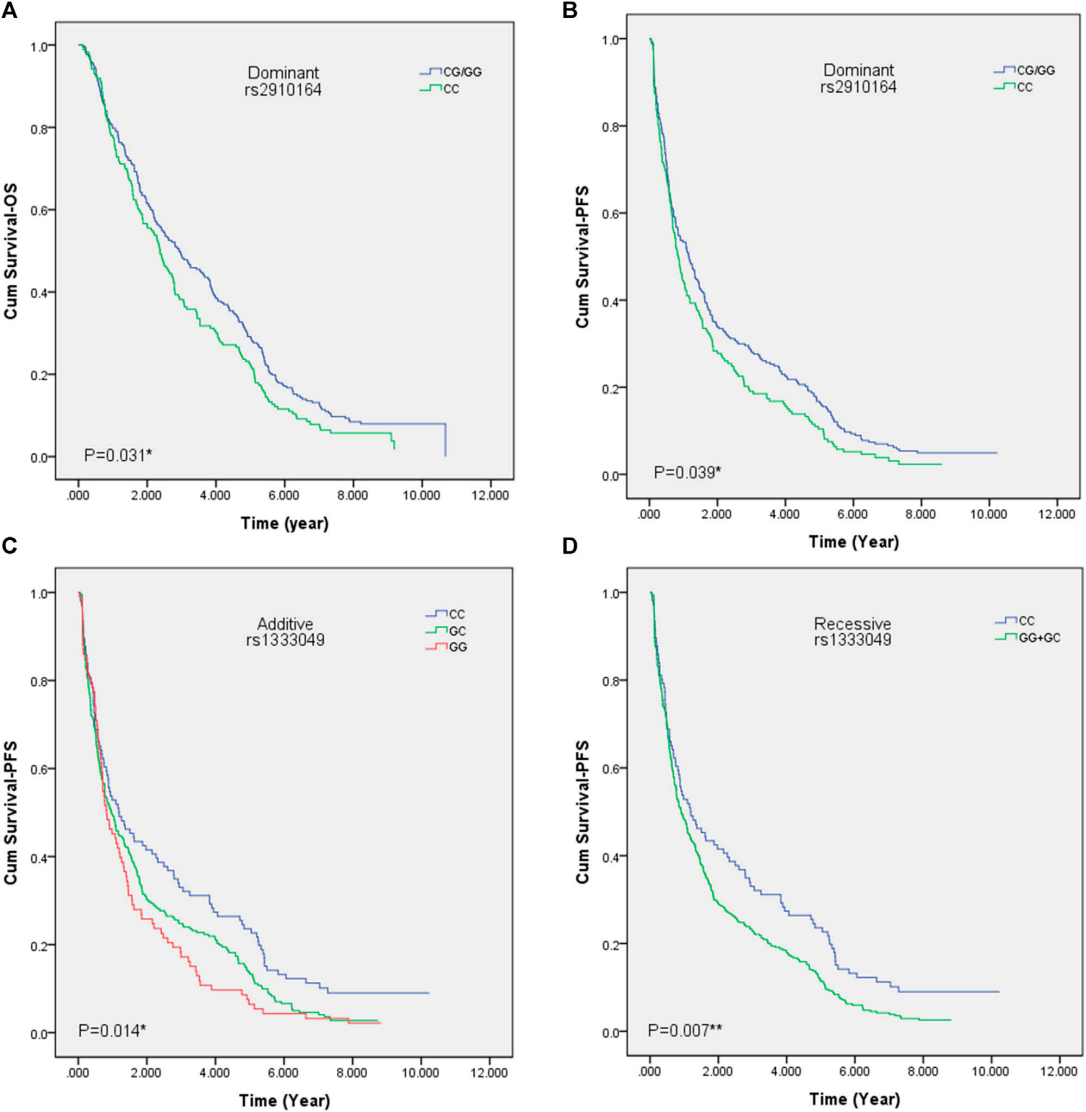
Kaplan Meier survival curves of patients with polymorphisms of *miR-146A* rs2910164 or *ANRIL* rs1333049. **(A)** Overall survival curve of patients with *miR-146A* rs2910164 SNP in dominant model. **(B)** Progression free survival curve of patients with *miR-146A* rs2910164 SNP in dominant model. **(C)** Progression free survival curve of patients with *ANRIL* rs1333049 SNP in additive model. **(D)** Progression free survival curve of patients with *ANRIL* rs1333049 SNP in recessive model. OS, overall survival; PFS, progression-free survival; *p < 0.05; **p < 0.01.

The genotyping result for the 22 ncRNA SNPs revealed a less than 95% call rate of *ANRIL* rs10120688, therefore it has been excluded from the following analyses. We focused our regression analysis on the rest to examine the relation between the 21 ncRNA SNPs and the prognosis of lung cancer patients. We found that *ANRIL* rs1333049 (G > C) was significantly associated with the PFS of lung cancer patients in the additive and recessive models [Additive model: HR = 0.84, *p* = 0.021, 95% CI (0.73–0.97); Recessive model: HR = 0.77, *p* = 0.026, 95% CI (0.61–0.97)]. In particular, patients with CC genotype of *ANRIL* rs1333049 showed a significantly longer median survival time of PFS (MST-PFS) compared to the patients with GC genotype, and the patients with GC genotype showed a longer MST-PFS compared to the patients with GG genotype (MST-PFS: 14.5 Vs 11.7 V s 10.1 months). In addition, patient with CC genotype of ANRIL rs1333049 also showed a significantly longer MST-PFS compared to the patients with GG or GC genotype (MST-PFS: 14.45 Vs 11.1 months). Patients carrying CC genotype in *ANRIL* rs1333049 showed lower risk of progression [HR = 0.71, *p* = 0.023, 95% CI (0.53–0.96)]. Patients carrying CG or GG genotype in *miR-146A* rs2910164 showed lower risk of death [HR = 0.81, *p* = 0.036, 95% CI (0.66–0.99)] and lower risk of progression [HR = 0.81, *p* = 0.040, 95% CI (0.67–0.99)] compared with CC genotype in the dominant model. In particular, patients who carry CG or GG genotype of miR-146A rs2910164 showed a significantly longer MST-OS and MST-PFS compared to the patients carrying CC genotype (MST-OS: 35.6 vs 28.8 months; MST-PFS: 14.1 vs 10.3 months). Additionally, the research result also illustrated that patients with CG genotype in *miRNA-146A* rs2910164 showed a lower risk of death [HR = 0.78, *p* = 0.023, 95% CI (0.63–0.97)] than patients with CC genotype. We also found that GA genotype in *miRNA-378A* rs1076064 can increase the risk of death [HR = 1.32, *p* = 0.026, 95% CI (1.03–1.69)] in contrast to the GG genotype. The remaining SNPs didn’t expose any association with either OS or PFS. The detailed results of our cox proportional hazard regression analysis are presented in Table 3, Supplementary Table S3, S4. Figure 1 summarizes the log-rank test and Kaplan Meier plots of the SNPs exhibiting statistical significance.

**TABLE 3:** Association of ncRNA polymorphisms with prognosis of patients with lung cancer (*n* = 446).

			OS			PFS	
Gene/SNP	Genotype/Genetic model	MST (month)	HR (95% CI)	P Value	MST (month)	HR (95% CI)	P Value
**miR-146A**	CC	28.8	1.00	—	10.3	1.00	—
**rs2910164**	CG	36.3	0.78 (0.63–0.97)	0.023*	14.1	0.82 (0.67–1.01)	0.062
	GG	32.5	0.91 (0.66–1.26)	0.569	13.9	0.78 (0.57–1.08)	0.130
	Additive	32.5/36.3/28.8	0.90 (0.77–1.05)	0.162	13.9/14.1/10.3	0.86 (0.74–1.00)	0.053
	Dominant	35.6/28.8	0.81 (0.66–0.99)	0.036*	14.1/10.3	0.81 (0.67–0.99)	0.040*
	Recessive	32.5/31.7	1.05 (0.78–1.42)	0.757	13.9/11.7	0.88 (0.65–1.18)	0.384
**miR-378A**	GG	36.1	1.00	—	11.1	1.00	—
**rs1076064**	GA	29.2	1.32 (1.03–1.69)	0.026*	11.4	1.04 (0.81–1.32)	0.783
	AA	37.1	0.99 (0.73–1.33)	0.920	16.2	0.89 (0.66–1.19)	0.423
	Additive	37.1/29.2/36.1	1.00 (0.87–1.15)	0.992	16.2/11.4/11.1	0.94 (0.81–1.09)	0.420
	Dominant	30.1	1.21 (0.96–1.54)	0.109	12.7	0.99 (0.78–1.25)	0.927
	Recessive	30.6	0.81 (0.63–1.04)	0.101	11.1	0.86 (0.68–1.10)	0.233
**ANRIL**	GG	33.5	1.00	—	10.1	1.00	—
**rs1333049**	GC	28.3	1.14 (0.89–1.46)	0.311	11.7	0.90 (0.71–1.15)	0.413
	CC	37.4	0.88 (0.65–1.18)	0.393	14.5	0.71 (0.53–0.96)	0.023*
	Additive	37.4/28.3/33.5	0.94 (0.81–1.08)	0.356	14.5/11.7/10.1	0.84 (0.73–0.97)	0.021*
	Dominant	32.1/33.5	1.05 (0.83–1.33)	0.700	12.7/10.1	0.84 (0.66–1.06)	0.148
	Recessive	37.4/29.5	0.80 (0.63–1.02)	0.068	14.45/11.1	0.77 (0.61–0.97)	0.026*

OS, overall survival; PFS, progression-free survival; MST, median survival time; HR, hazard ratio; CI, confidence interval; *p < 0.05.

### Meta-Analysis Confirming the Association of *miR-146A* rs2910164 and Prognosis of Lung Cancer

**FIGURE 2:**
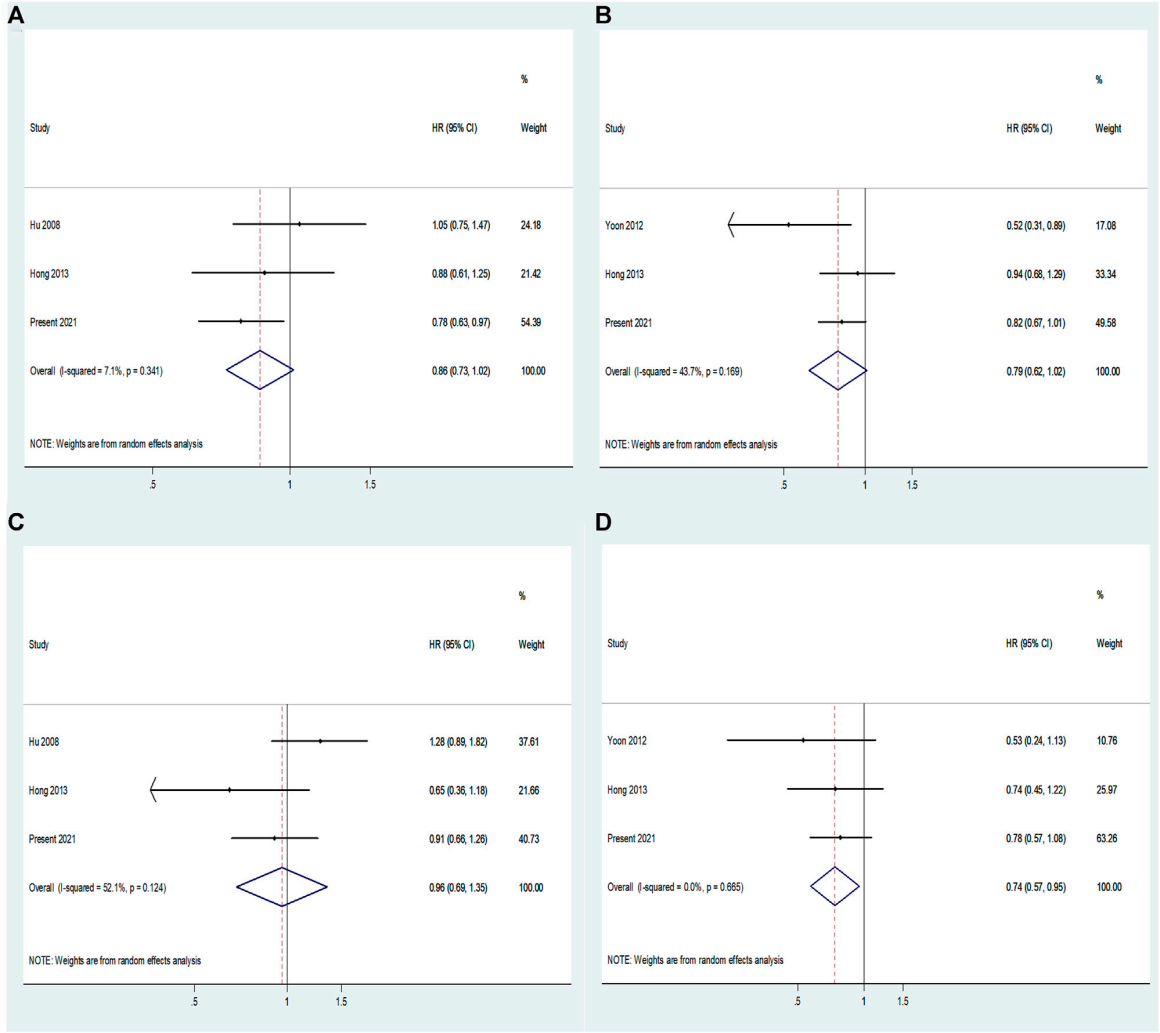
Forest plots of the association between *miR-146A* rs2910164 and prognosis of lung cancer. **(A)** Overall survival: forest plot of *miR-146A* rs2910164 in CG vs GG. **(B)** Progression-free survival: forest plot of *miR-146A* rs2910164 in CG vs GG. **(C)** Overall survival: forest plot of *miR-146A* rs2910164 in GG vs CC. **(D)** Progression-free survival: forest plot of *miR-146A* rs2910164 in GG vs CC.

We did not identify any previous study addressing the correlation between *ANRIL* rs1333049 and the prognosis of lung cancer, but we found three correlation studies between *miR-146A* rs2910164 and the prognosis of lung cancer (Hu et al., 2008; Yoon et al., 2012; Hong et al., 2013). We conducted meta-analyses (Figure 2) for these three studies and our results. The populations in these studies were all from East Asia, which is similar to the ethnic origin of the population in our study. The population in these studies received various treatments, including surgery, chemotherapy and radiotherapy, while the treatment regimen in our study was limited to chemotherapy. *miR-146A* rs2910164 in CG vs GG exhibited low and moderate degree of heterogeneity in the association analyses on OS and PFS. This analytical result indicated that CG has a lower risk of death and progression compared to GG genotype (Figures 2A,B). Respectively, *miR-146A* rs2910164 in GG vs CC exhibited high and low degrees of heterogeneity in the association analyses on OS and PFS (Figures 2C,D). In particular, the meta-analysis indicated that GG has a lower risk of progression compared to CC, and the genotypes of GG and CC did not show a significant difference in the association with OS. The above meta-analysis confirmed that miR-146A rs2910164 is associated with a better prognosis of lung cancer.

### Stratification Analysis of Association of Two Non-CodingRNA Single Nucleotide Polymorphisms and Prognosis in Lung Cancer Patients Treated With Platinum-Based Chemotherapy

**FIGURE 3:**
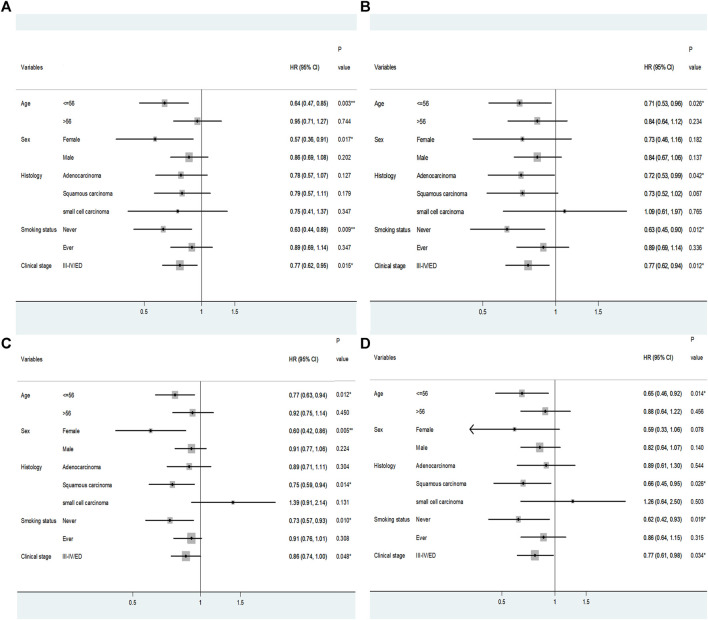
Stratification analysis for the association between **(A)** *miR-146A* rs2910164, **(B)** *ANRIL* rs1333049 and OS/PFS in additive or recessive model. The center of rhombus represents the HR value, and the width range of the horizontal line represents 95% CI. *p < 0.05, **p < 0.01; ED, extensive-stage disease; OS, overall survival; PFS, progression-free survival.

Additional detailed analyses have been performed to investigate how the two SNPs of *ANRIL* rs1333049 and *miR-146A* rs2910164 are associated with the prognosis of lung cancer patients. The stratification analysis regarding age, sex, histology, smoking status and clinical stage is shown in Figure 3. This analysis was only applied to the group of advanced stage, since the samples of the group “I-II/LD” are limited. In terms of the younger patients (≤56 years old), the CG or GG genotype in rs2910164 exhibited distinct lower risk of death [HR = 0.64, *p* = 0.003, 95% CI (0.47–0.85)] and lower risk of progression [HR = 0.71, *p* = 0.026, 95% CI (0.53–0.96)] versus the CC genotype. Among never smoking patients, the CG or GG genotype in rs2910164 demonstrated lower risk of death [HR = 0.63, *p* = 0.009, 95% CI (0.44–0.89)] and lower risk of progression [HR = 0.63, *p* = 0.012, 95% CI (0.45–0.90)] compared to CC genotype. When advanced clinical stage was presented, observable lower risk of death [HR = 0.77, *p* = 0.015, 95% CI (0.62–0.95)] and lower risk of progression [HR = 0.77, *p* = 0.012, 95% CI (0.62–0.94)] occurred to patients carrying CG or GG genotype in contrast to CC genotype in rs2910164. In female subgroup, *ANRIL* rs1333049 genotype presents lower risk of progression [HR = 0.60, *p* = 0.005, 95% CI (0.42–0.86)] in additive model. In terms of the younger patients (≤56 years old), rs1333049 genotype showed lower risk of progression in additive and dominant models [Additive model: HR = 0.77, *p* = 0.012, 95% CI (0.63–0.94); recessive model: HR = 0.65, *p* = 0.014, 95% CI (0.46–0.92)]. In terms of squamous carcinoma, rs1333049 genotype showed lower risk of progression in additive and recessive models [Additive model: HR = 0.75, *p* = 0.014, 95% CI (0.59–0.94); recessive model: HR = 0.66, *p* = 0.026, 95% CI (0.45–0.95)]. In the subgroup of never smoking patients, rs1333049 genotype showed lower risk of progression in additive and recessive models [Additive model: HR = 0.73, *p* = 0.010, 95% CI (0.57–0.93); recessive model: HR = 0.62, *p* = 0.019, 95% CI (0.42–0.93)].

## Discussion

Precision medicine has advanced prognostic prediction with the support of molecular biomarkers. In order to identify the biomarkers associated with the prognosis of lung cancer under platinum-based chemotherapy, we aimed to explore the SNPs of ncRNA. Recent studies uncovered how non-coding RNAs function in the initiation and proliferation processes of cancer, which has changed the landscape of this field. Many miRNAs and lncRNAs were reported to be possible tumor suppressor factors or oncogenic factors for lung cancer (Liz and Esteller, 2016). Since the biological function of the variations in miRNA or lncRNA could be affected by a changed expression, secondary structures, and binding region (Ryan et al., 2010), thus theoretically these variations may have an impact on the initiation, proliferation and prognosis of tumor. Our study genotyped 22 SNPs of miRNA or lncRNA for lung cancer patients and confirmed the potential association between several SNPs and the prognosis of lung cancer.

MiR-146a has been identified as a tumor suppressor and immune regulator (Okada et al., 2010). For human lung adenocarcinoma cell lines, it may be involved in the processes of proliferation and migration (Yuan et al., 2020). A related meta-analysis suggested that miR-146a could be adopted as a prognostic biomarker for NSCLC, which means detection of overexpressed miR-146a may lead to a better prognosis (Zhan et al., 2016). Several other studies, including our previous study, have also proved that *miR-146A* rs2910164 (G > C) is a widely recognized risk factor of lung cancer (Yin et al., 2017; Hao et al., 2018). Based on 6,506 cases and 6,576 controls collected from fifteen studies, a meta-analysis highlighted the conclusion that *miR-146A* rs2910164 (G > C) polymorphism has a notable association with the susceptibility of lung cancer (Wang et al., 2020). Meanwhile, another study showed *miR-146a* rs2910164 (C > G) is associated with a better prognosis of completely resected non-small cell lung cancer (Yoon et al., 2012). The potential reason is that the presence of the C allele in *pre-miR-146a* decreases the expression of mature miRNA (Jazdzewski et al., 2008). These previous studies have confirmed that *miR-146a* rs2910164 (G > C) is a risk factor for the lung cancer susceptibility and worse prognosis of completely resected NSCLC. In this study, we revealed that rs2910164 (C > G) leads to a better prognosis of the lung cancer patients treated with platinum-based chemotherapy.

MiR-378A is reportedly a tumor suppressor in gastric cancer and down-regulated in gastric cancer (Deng et al., 2013). A study by Wang et al. showed the cell proliferation could be suppressed by miR-378A when CDC40 is selected to be the target (Wang et al., 2014). In terms of colorectal cancer, miR-378A has a suppressive impact on the growth of cells, and increasing of L-OHP-induced apoptosis may also occur as a result (Wang et al., 2014). Another study in hepatocellular carcinoma has highlighted the connection between the G allele of *miR-378A* rs1076064 and better prognosis (An et al., 2014). Meanwhile, our study found that the GA genotype is associated with worse prognosis compared with the GG genotype in lung cancer. Furthermore, a study by An et al. showed that rs1076064 belongs to the flanking region of *pre-miR-378A* that is involved with promoter activity, and the G allele of rs1076064 can intensify the promoter activity, therefore, strengthen the expression of *miR-378A* (An et al., 2014). This could explain the association between rs1076064 and the prognosis of various cancers.

ANRIL is able to regulate the tumor suppressors CDKN2A/B. In particular, its downregulation could increase apoptosis and inhibit cell proliferation, migration, and invasion in lung cancer cell lines (Nie et al., 2015). Besides, ANRIL is overexpressed in NSCLC tissues and associated with clinical stage and prognosis (Nie et al., 2015). *ANRIL* rs1333049 is an intergene variant in the cyclin-dependent kinase inhibitor 2A/B (CDKN2A/B). Its relationship to cardiovascular disease is well described, but rarely described in tumor field research. An American study determined that the G allele of rs1333049 is a risk allele for worse prognosis in breast cancer (Cleveland et al., 2012). However, another two studies claimed that the C allele of rs1333049 is associated with poor prognosis in esophageal squamous cell carcinoma and breast cancer respectively (Ghobadi et al., 2019; Seifi et al., 2020). Our research indicated the connection between the G allele of rs1333049 and poor prognosis of lung cancer. Furthermore, another study found that GG genotype is associated with the highest expression of *ANRIL* in vascular smooth muscle cells (Motterle et al., 2012). Therefore, presumably GG genotype may cause overexpression of *ANRIL* in lung cancer tissues as well. Such overexpression may accelerate the proliferation of tumor, and therefore influence the prognosis of lung cancer. This could explain the association between the G allele of rs1333049 and poor prognosis in lung cancer, though the underlying mechanism has yet to be verified by additional functional studies.

This study is limited by the sample size. Also, these samples are collected from the same clinical environment within a certain geographic region. To generalize the outcome of our study, we are collecting additional patient samples to create a larger independent dataset for extended validation. Furthermore, the validated SNPs will be examined by functional verification, assuring their effectiveness regarding the prognosis of lung cancer patients treated with platinum-based chemotherapy.

In conclusion, our research identified a significant association between two SNPs, namely *ANRIL* rs1333049 and *miR-146A* rs2910164, and the prognosis of lung cancer patients under platinum-based chemotherapy. In this study, both *ANRIL* rs1333049 (G > C) and *miR-146A* rs2910164 (C > G) showed a better prognosis for these lung cancer patients. Therefore, we suggest these two SNPs could be used as biomarkers to facilitate the prediction of lung cancer prognosis.

## Data Availability

The original contribution presented in the study are included in the article/Supplementary Material, further inquiries can be directed to the corresponding authors.
