# Supplementary material for: Non-Coding RNA Polymorphisms (rs2910164 and rs1333049) Associated With Prognosis of Lung Cancer Under Platinum-Based Chemotherapy
Source: Front Pharmacol. 2021 Sep 16;12:709528. doi: 10.3389/fphar.2021.709528 (PMC8481925; doi:10.3389/fphar.2021.709528)
Supplement: Supplementary file 1 [file Table1.DOCX]

**Table S1.** The information of the 22 selected SNPs in ncRNA.

| **Gene** | **SNP ID** | **PMID** | **MAF** | **CADD score** | **Polymorphism** | **Function** ^a)^ | **Tag SNP/ located in LD** |
| --- | --- | --- | --- | --- | --- | --- | --- |
| *mir-196a-2* | rs11614913 | 23470291 | 0.33 | 19.48 | C>T | expression decreases | Yes |
| *mir-146a* | rs2910164 | 23470291 | 0.39 | 17.14 | C>G | expression increases (PMID: 25154761) | No |
| *mir-5197* | rs2042253 | 25103824 | 0.31 | 6.59 | T>C | expression change mildly | Yes |
| *mir-378* | rs1076064 | 30619739 | 0.49 | 17.03 | A>G | expression increases (PMID: 24751683) | No |
| *mir-27a* | rs895819 | 29100439 | 0.36 | 17.16 | T>C | expression change mildly | Yes |
| *mir-149* | rs71428439 | 26550305 | 0.14 | 0.26 | A>G | expression change mildly | Yes |
| *mir-30c-1* | rs928508 | 29100439 | 0.45 | 17.97 | G>A | expression increases (PMID: 20889907) | No |
| *mir-605* | rs2043556 | 31938129 | 0.26 | 3.07 | T>C | expression increases | No |
| *let-7a-2* | rs629367 | 26625972 | 0.15 | 2.93 | C>A | expression decreases (PMID: 24760009) | No |
| *mir-218-1* | rs11134527 | 29692628 | 0.35 | 19.00 | G>A | expression decreases (PMID: 23566829) | No |
| *mir-499* | rs3746444 | 23470291 | 0.18 | 19.25 | A>G | target gain/loss | Yes |
| *H19* | rs2839698 | 26729200 | 0.29 | 5.15 | G>A | impact lncRNA:miRNA interaction | Yes |
| *H19* | rs2107425 | 26729200 | 0.45 | 8.07 | C>T | expression decreases (PMID: 31769935) | No |
| *MALAT1* | rs619586 | 29802154 | 0.07 | 4.65 | A>G | impact lncRNA:miRNA interaction | Yes |
| *HOTAIR* | rs7958904 | 27330313 | 0.48 | 0.42 | C>G | impact lncRNA:miRNA interaction and effect structure of lncRNA | Yes |
| *HOTAIR* | rs4759314 | 30464618 | 0.1 | 16.80 | G>A | expression decreases (PMID: 23566829) | No |
| *MEG3* | rs116907618 | 28260796 | 0.06 | 7.08 | G>C | impact lncRNA:miRNA interaction | No |
| *HOTTIP* | rs3807598 | 30940774 | 0.34 | 6.37 | C>G | impact lncRNA:miRNA interaction | Yes |
| *HOTTIP* | rs1859168 | 29802154 | 0.13 | 5.42 | A>C | impact lncRNA:miRNA interaction | Yes |
| *CCAT2* | rs6983267 | 22848662 | 0.39 | 17.85 | G>T | impact lncRNA:miRNA interaction | Yes |
| *ANRIL* | rs10120688 | 26729200 | 0.42 | 2.10 | G>A | impact lncRNA:miRNA interaction and effect structure of lncRNA | Yes |
| *ANRIL* | rs1333049 | 31721206 | 0.42 | 0.32 | G>C | expression increases (PMID: 19592466) | No |

MAF, minor allele frequency (Ensembl); CADD, combined annotation dependent depletion; ^a)^Function, no PMID: prediction from miRNASNP v3 or lncRNASNP2.
