# Supplementary figures and images for: Non-Coding RNA Polymorphisms (rs2910164 and rs1333049) Associated With Prognosis of Lung Cancer Under Platinum-Based Chemotherapy
Source: Front Pharmacol. 2021 Sep 16;12:709528. doi: 10.3389/fphar.2021.709528 (PMC8481925; doi:10.3389/fphar.2021.709528)

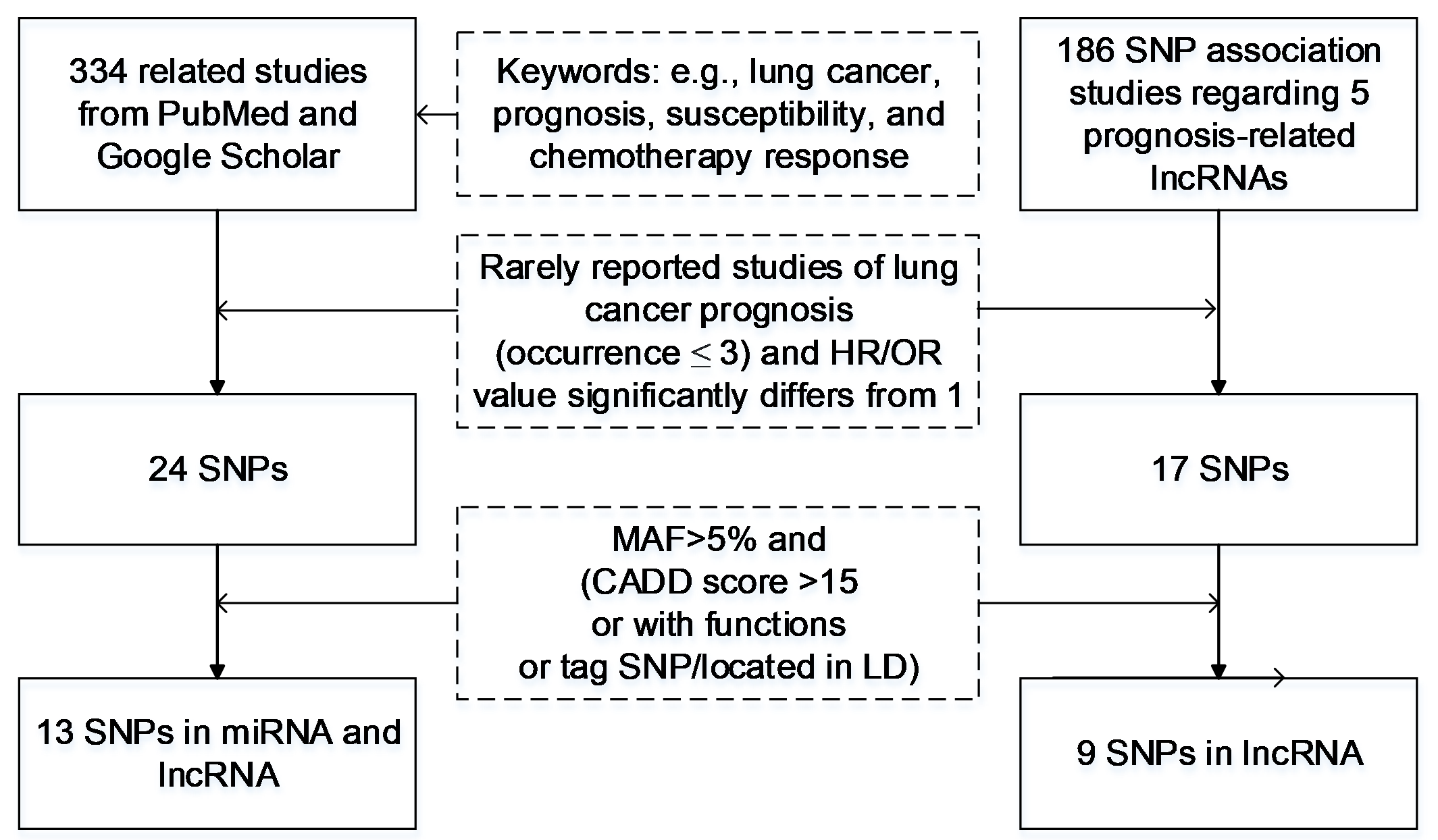

Supplement: Supplementary file 2 [file Image1.TIF]
