# Supplementary material for: Non-Coding RNA Polymorphisms (rs2910164 and rs1333049) Associated With Prognosis of Lung Cancer Under Platinum-Based Chemotherapy
Source: Front Pharmacol. 2021 Sep 16;12:709528. doi: 10.3389/fphar.2021.709528 (PMC8481925; doi:10.3389/fphar.2021.709528)
Supplement: Supplementary file 3 [file Table2.DOCX]

**Table S2.** Primers of all selected SNPs.

| **SNP ID** | **2nd-PCRP** | **1st-PCRP** |
| --- | --- | --- |
| rs2042253 | ACGTTGGATGCAGTCTCTTCTTGAACTGGC | ACGTTGGATGCTGATCTACAGCTAGGGAAG |
| rs2043556 | ACGTTGGATGGAGAGCAATATACCTGTGGC | ACGTTGGATGAACAGAGAAGGCACTATGAG |
| rs2910164 | ACGTTGGATGAAGCCGATGTGTATCCTCAG | ACGTTGGATGCACGATGACAGAGATATCCC |
| rs3746444 | ACGTTGGATGGGAAGCAGCACAGACTTG | ACGTTGGATGGGCTGTTAAGACTTGCAGTG |
| rs71428439 | ACGTTGGATGTCTTCACTCCCGTGCTTGTC | ACGTTGGATGGCCCGGCGACCTGCGTTGT |
| rs928508 | ACGTTGGATGAAGAGTGCTGCCTATTTGGG | ACGTTGGATGCTGAGAACAGTGCTAATTGC |
| rs11614913 | ACGTTGGATGCTGATCTGTGGCTTAGGTAG | ACGTTGGATGTCGACGAAAACCGACTGATG |
| rs895819 | ACGTTGGATGACTTAGCCACTGTGAACACG | ACGTTGGATGAGCAGGGCTTAGCTGCTTGT |
| rs629367 | ACGTTGGATGTATGCAGCATTTTTGTGAC | ACGTTGGATGATTCTGTTTCCTCGGGTTAG |
| rs1076064 | ACGTTGGATGAGATCACCAGAAGATCCTCG | ACGTTGGATGGCAGTGAAAGTTAATCTGGG |
| rs11134527 | ACGTTGGATGAGAGGAAGCAGCGTGGAGAA | ACGTTGGATGAAGTGTTCCAGTGGAACCCC |
| rs2839698 | ACGTTGGATGTTGGACATCTGGAGTCTGGC | ACGTTGGATGAAAGTGACCGGGATGAATGC |
| rs2107425 | ACGTTGGATGGGTTGTAGTTGTGGAATCGG | ACGTTGGATGATGCCCGACCTGAAGATCTG |
| rs619586 | ACGTTGGATGCAAAACCCCCGGAACTTTTA | ACGTTGGATGTTTGCCACTTCTCAACCGTC |
| rs7958904 | ACGTTGGATGTCTGGTCTTGTTAACAAGCC | ACGTTGGATGCAGAGAACGCTGGAAAAACC |
| rs4759314 | ACGTTGGATGGCATTTTAAGTGTTCCTGCC | ACGTTGGATGTATCTGAGGGAGTAAGCCTG |
| rs116907618 | ACGTTGGATGCTCCTTCTCCCTAACAAACG | ACGTTGGATGTTCCTTCCTCAGAGTTTGCC |
| rs3807598 | ACGTTGGATGTGTGCGAATGTGAGTGTGTC | ACGTTGGATGTATCGGTTCCTGCGGATATG |
| rs1859168 | ACGTTGGATGAATGATAGGGACACATCGGG | ACGTTGGATGAGGTTTGTCTGAGAGGGATG |
| rs6983267 | ACGTTGGATGTCATCGTCCTTTGAGCTCAG | ACGTTGGATGATACACAGCCCAGTCTAAGG |
| rs10120688 | ACGTTGGATGGAAGCCAGAAAAGAAGACCG | ACGTTGGATGGCCACATAGATTAGAGGCAG |
| rs1333049 | ACGTTGGATGATGTGACTGCTTCTGCATAC | ACGTTGGATGGTGTATGACACTTCTTAGGC |
