# Supplementary material for: Non-Coding RNA Polymorphisms (rs2910164 and rs1333049) Associated With Prognosis of Lung Cancer Under Platinum-Based Chemotherapy
Source: Front Pharmacol. 2021 Sep 16;12:709528. doi: 10.3389/fphar.2021.709528 (PMC8481925; doi:10.3389/fphar.2021.709528)
Supplement: Supplementary file 4 [file Table3.DOCX]

**Table S3.** Association of miRNA polymorphisms with prognosis of patients with lung cancer (n=446).

|  |  | **OS** | | **PFS** | |
| --- | --- | --- | --- | --- | --- |
| **Gene/SNP** | **Genetic model** | **HR (95% CI)** | **P value** | **HR (95% CI)** | **P value** |
| *miR-605* | Additive | 1.13 (0.96-1.32) | 0.154 | 1.02 (0.87-1.20) | 0.816 |
| rs243556 | Dominant | 1.15 (0.93-1.41) | 0.191 | 1.02 (0.83-1.24) | 0.887 |
|  | Recessive | 1.19 (0.82-1.73) | 0.365 | 1.06 (0.73 -1.53) | 0.778 |
| *miR-149* | Additive | 0.98 (0.81-1.18) | 0.803 | 1.14 (0.94-1.37) | 0.190 |
| rs71428439 | Dominant | 1.02 (0.81-1.28) | 0.868 | 1.21 (0.96-1.52) | 0.103 |
|  | Recessive | 0.70 (0.37-1.32) | 0.272 | 0.95 (0.52-1.74) | 0.872 |
| *miR-196a-2* | Additive | 0.96 (0.84-1.11) | 0.602 | 1.00 (0.87-1.15) | 0.994 |
| rs11614913 | Dominant | 1.02 (0.82-1.27) | 0.850 | 0.96 (0.77-1.19) | 0.696 |
|  | Recessive | 0.87 (0.69-1.11) | 0.273 | 1.06 (0.83-1.34) | 0.650 |
| *miR-27a* | Additive | 1.14 (0.98-1.33) | 0.091 | 1.10 (0.95-1.28) | 0.207 |
| rs895819 | Dominant | 1.24 (0.87-1.76) | 0.234 | 1.22 (0.87-1.71) | 0.257 |
|  | Recessive | 1.16 (0.96-1.41) | 0.133 | 1.10 (0.91-1.34) | 0.331 |
| *miR-499* | Additive | 0.97 (0.79-1.21) | 0.803 | 0.97 (0.79-1.19) | 0.771 |
| rs3746444 | Dominant | 0.95 (0.76-1.19) | 0.639 | 0.97 (0.78-1.21) | 0.779 |
|  | Recessive | 1.60 (0.65-3.91) | 0.304 | 0.94 (0.39-2.30) | 0.895 |
| *let-7a-2* | Additive | 1.05 (0.89-1.24) | 0.597 | 0.97 (0.83-1.14) | 0.730 |
| rs629367 | Dominant | 1.08 (0.88-1.32) | 0.469 | 1.01 (0.83-1.23) | 0.939 |
|  | Recessive | 0.96 (0.61-1.51) | 0.861 | 0.79 (0.50-1.24) | 0.301 |
| *miR-30c-1* | Additive | 0.91 (0.78-1.05) | 0.205 | 0.95 (0.82-1.09) | 0.448 |
| rs928508 | Dominant | 0.90 (0.71-1.15) | 0.398 | 0.92 (0.73-1.17) | 0.503 |
|  | Recessive | 0.87 (0.68-1.10) | 0.240 | 0.94 (0.74-1.19) | 0.579 |
| *miR-218-1* | Additive | 0.96 (0.83-1.10) | 0.537 | 0.94 (0.82-1.09) | 0.423 |
| rs11134527 | Dominant | 0.91 (0.74-1.12) | 0.370 | 0.87 (0.71-1.06) | 0.167 |
|  | Recessive | 1.00 (0.77-1.30) | 1.000 | 1.04 (0.80-1.35) | 0.768 |
| *miR-5197* | Additive | 1.05 (0.90-1.22) | 0.515 | 1.04 (0.89-1.20) | 0.638 |
| rs2042253 | Dominant | 1.05 (0.85-1.29) | 0.680 | 1.05 (0.85-1.28) | 0.679 |
|  | Recessive | 1.11 (0.83-1.47) | 0.496 | 1.05 (0.79-1.40) | 0.742 |

OS, overall survival; PFS, progression-free survival; HR, hazard ratio; CI, confidence interval.
