# Supplementary material for: Non-Coding RNA Polymorphisms (rs2910164 and rs1333049) Associated With Prognosis of Lung Cancer Under Platinum-Based Chemotherapy
Source: Front Pharmacol. 2021 Sep 16;12:709528. doi: 10.3389/fphar.2021.709528 (PMC8481925; doi:10.3389/fphar.2021.709528)
Supplement: Supplementary file 5 [file Table4.DOCX]

**Table S4.** Association of lncRNA polymorphisms with prognosis of patients with lung cancer (n=446).

|  |  | **OS** | | **PFS** | |
| --- | --- | --- | --- | --- | --- |
| **Gene/SNP** | **Genotype/**  **Genetic model** | **HR (95% CI)** | **P value** | **HR (95% CI)** | **P value** |
| *H19* | Additive | 1.13 (0.97-1.32) | 0.125 | 1.04 (0.89-1.22) | 0.598 |
| rs2839698 | Dominant | 1.11 (0.91-1.35) | 0.301 | 1.00 (0.82-1.21) | 0.994 |
|  | Recessive | 1.37 (0.96-1.96) | 0.082 | 1.27 (0.89-1.81) | 0.187 |
| *H19* | Additive | 0.95 (0.82-1.09) | 0.430 | 0.97 (0.85-1.12) | 0.713 |
| rs2107425 | Dominant | 1.03 (0.84-1.26) | 0.802 | 1.02 (0.84-1.25) | 0.828 |
|  | Recessive | 0.76 (0.57-1.02) | 0.065 | 0.86 (0.65-1.15) | 0.313 |
| *MALAT1* | Additive | 0.92 (0.72-1.18) | 0.527 | 0.93 (0.73-1.18) | 0.542 |
| rs619586 | Dominant | 0.91 (0.70-1.18) | 0.486 | 0.94 (0.73-1.22) | 0.653 |
|  | Recessive | 1.07 (0.34-3.35) | 0.908 | 0.63 (0.20-1.97) | 0.428 |
| *HOTAIR* | Additive | 0.98 (0.83-1.14) | 0.748 | 0.96 (0.82-1.12) | 0.586 |
| rs7958904 | Dominant | 0.99 (0.81-1.20) | 0.895 | 0.96 (0.79-1.16) | 0.672 |
|  | Recessive | 0.90 (0.61-1.33) | 0.599 | 0.91 (0.62-1.33) | 0.906 |
| *HOTAIR* | AA | 1.00 |  | 1.00 |  |
| rs4759314 | GA | 1.17 (0.88-1.56) | 0.227 | 1.16 (0.87-1.55) | 0.304 |
| *MEG3* | Additive | 1.19 (0.93-1.52) | 0.165 | 1.26 (0.98-1.61) | 0.068 |
| rs116907618 | Dominant | 1.21 (0.92-1.58) | 0.170 | 1.24 (0.95-1.62) | 0.109 |
|  | Recessive | 1.31 (0.48-3.52) | 0.599 | 2.24 (0.83-6.07) | 0.113 |
| *HOTTIP* | Additive | 1.04 (0.90-1.21) | 0.578 | 1.06 (0.91-1.23) | 0.460 |
| rs3807598 | Dominant | 1.03 (0.81-1.30) | 0.807 | 1.02 (0.81-1.29) | 0.841 |
|  | Recessive | 1.09 (0.85-1.39) | 0.508 | 1.13 (0.89-1.44) | 0.309 |
| *HOTTIP* | Additive | 1.08 (0.93-1.25) | 0.324 | 1.11 (0.95-1.29) | 0.177 |
| rs1859168 | Dominant | 1.06 (0.85-1.30) | 0.618 | 1.04 (0.84-1.28) | 0.731 |
|  | Recessive | 1.18 (0.90-1.55) | 0.233 | 1.34 (1.02-1.75) | 0.054 |
| *CCAT2* | Additive | 1.01 (0.88-1.17) | 0.865 | 0.94 (0.82-1.07) | 0.336 |
| rs6983267 | Dominant | 1.03 (0.80-1.33) | 0.838 | 0.94 (0.73-1.19) | 0.591 |
|  | Recessive | 1.01 (0.81-1.25) | 0.932 | 0.90 (0.72-1.11) | 0.308 |

OS, overall survival; PFS, progression-free survival; HR, hazard ratio; CI, confidence interval.
